# Supplementary material for: A pattern of platelet indices as a potential marker for prediction of pre-eclampsia among pregnant women attending a Tertiary Hospital, Ethiopia: A case-control study
Source: PLoS One. 2021 Nov 9;16(11):e0259543. doi: 10.1371/journal.pone.0259543 (PMC8577759; doi:10.1371/journal.pone.0259543)
Supplement: Attachment — Submitted filename: Responses for reviwers and editors.docx [file pone.0259543.s001.docx]

**Rebuttal Letter**
**Responses for Editors**

1. Our manuscript meets PLOS ONE's guideline style requirements

2. We tried to correct manuscript spelling and grammar: revised and corrected [we tried to show for free sample editors like paper true, and for our colleagues and online Grammarly].

3. Sorry, we haven’t received any grant and grant number and it should be covered by the institution for data collectors and supervisors for data collection of research article.

We tried to make similar Funding Information’ and ‘Financial Disclosure’ sections in the cover letter online submission: updated and revised

4. The Acknowledgments Section of our manuscript and funding information which should be appeared in the Acknowledgments section or other areas of our manuscript were removed: corrected and revised

In our cover letter amended should be made; corrected and revised

5. Competing Interests section: corrected and revised. 
6. Our Data Availability statement corrected and revised.

There are no ethical or legal restrictions to sharing your data publicly,

The authors not be the sole named individuals responsible for ensuring data access.

Our Data Availability statement should be provided in our cover letter.

7. Removed

8. ORCID ID for the corresponding author validated in Editorial Manager. Updated.

9. Authors’ affiliations should reflect the institution where the work was done: updated

**Responses For Reviewers' Comments:**
1. Reviewer #1: Our the manuscript technically sound, and do the data support the conclusions Reviewer #2: Our the manuscript technically sound, and data support the conclusions

2. Reviewer #1: the statistical analysis been performed appropriately and rigorously.

Reviewer #2: the statistical analysis been performed appropriately and rigorously.

3. Reviewer #1: all data underlying the findings in their manuscript fully available Reviewer #2: all data underlying the findings in their manuscript fully available

4. Reviewer #1: the manuscript presented in an intelligible fashion and written in Standard English we tried to correct all typographical errors by showing for different experts.

Reviewer #2: the manuscript presented in an intelligible fashion and written in Standard English we tried to correct all typographical errors by showing for different experts. See sample attached as supporting information.
5. Reviewer #1: **Title**: corrected and revised.

2) **Abstract:**
1. The practice of platelet indices in the diagnosis of preeclampsia is not yet extensive in Ethiopia and there is little information on platelet patterns in preeclampsia and normal pregnancy!!! As my literature search, for future platetelate indices are proposed to replace proteinuria in diagnosis of preeclampsia, but using its parameters as diagnostic tool is not as such growing in Ethiopia: corrected and revised.FOR further information visit this reference[Poon LC, Nicolaides KH. Early prediction of preeclampsia. Obstet Gynecol Int. 2014; 2014:297397 and Sahin S, Ozakpinar O, Eroglu M, Tetik S. Platelets in Preeclampsia: Function and Role in the Inflammation. Journal of Marmara University Institute of Health Sciences. 2014:1] and the statement were rephrased.
2. The purpose of this study was: corrected and revised.
3) **Patients and Methods:**
1 the preeclampsia, mild and sever eclampsia defined in separate page with the references: revised and corrected.

BMI: defined and revised.
2. Reference was added and the normal and abnormal platelet indices: corrected and revised in operational definition parts try to get it in clean munscrupit.
3. We have estimated the platelet indices once on preeclamptic and normal pregnant women methods section: already indicated in methods section

4) **Result section:**
1. our design of the study :corrected and revised

2. We categorized our cases to severer and non-sever according to guideline adding a reference to them and we make it the same words in the text and in the tables: revised and corrected
3. Footnotes for all the tables with the full name of the abbreviations: corrected and revised.

Reviewer2 #we tried to correct and revise the grammatical errors that compromise understanding of the methodology and findings of this munscrupit. We tried to correct manuscript spelling and grammar

6. We need to be published the peer review (our review may still be made public).
Our figure files to the Pre-flight Analysis and Conversion Engine (PACE) digital diagnostic tool, revised and corrected (updated).
